# Co‐Encapsulation of Purslane Seed Oil and Tarragon Essential Oil and Investigating the Use of It as a Natural Preservative and Functional Compound in Burger Formulation

**DOI:** 10.1002/fsn3.71513

**Published:** 2026-03-13

**Authors:** Mozhgan Mehrabi, Homa Baghaei, Majid Mohammadhosseini, Fariborz Nahidi

**Affiliations:** ^1^ Department of Food Science & Technology, Da.C., Islamic Azad University Damghan Iran; ^2^ Department of Chemistry and Biochemistry, Sha.C., Islamic Azad University Shahrood Iran; ^3^ Herbal Drugs Raw Materials Research Center (HDRMRC), Sha.C., Islamic Azad University Shahrood Iran

**Keywords:** burger, fatty acid profile, fortification, natural preservative, purslane oil, tarragon

## Abstract

The study aimed to evaluate the application of purslane seed oil–tarragon essential oil (PO–TE) nanocapsules as natural preservatives and functional elements in beef burger production. The nanocapsules were created by combining different amounts of gum arabic (GA) with whey protein isolate (WPI) to achieve desired emulsifying and film‐forming and oxidative protection effects from polysaccharide–protein biopolymer systems. Among the tested formulations (GA0:WPI1, GA1:WPI0, GA3:WPI1, GA1:WPI1, and GA1:WPI3), the selected nanocapsule was partially substituted for fat in burger formulations at levels of 0%, 0.9%, 1.8%, and 2.7%, and product quality was evaluated during 12 days of refrigerated storage. The GA3:WPI1 nanocapsule showed excellent physicochemical stability through its −33.0 mV zeta potential and strong antioxidant activity (DPPH radical scavenging activity, 2,2‐diphenyl‐1‐picrylhydrazyl assay) and high encapsulation efficiency. The study revealed that adding PO–TE nanocapsules to burgers enhanced their oxidative stability and decreased both lipid oxidation markers and microbial growth rates at various concentrations (*p* < 0.05). The nanocapsules prevented negative changes in pH and total volatile base nitrogen and color and texture and sensory attributes during storage while decreasing cooking loss. The use of PO–TE nanocapsules in burgers resulted in an increased amount of polyunsaturated fatty acids which caused a nutritional transformation that decreased the amount of saturated fatty acids. Overall, the results demonstrate that PO–TE nanocapsules represent a promising formulation strategy for developing clean‐label meat products with improved shelf life, nutritional value, and functional performance.

## Graphical abstract

This study developed purslane seed oil–tarragon essential oil (PO–TE) nanocapsules using gum Arabic (GA) and whey protein isolate (WPI) as wall materials. The co‐encapsulation significantly enhanced oxidative stability, antimicrobial activity, and polyunsaturated fatty acid retention in beef burgers during refrigerated storage. Incorporation of PO–TE nanocapsules into burger formulation effectively reduced lipid oxidation, improved sensory quality, and extended shelf life, demonstrating their potential as a natural preservative and functional ingredient in meat products.

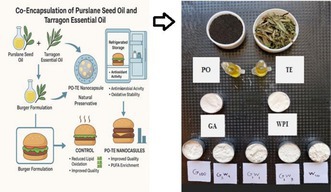

## 1 Introduction

Nowadays, due to increasingly busy lifestyles, consumer demand for fast foods has risen substantially, and burgers remain among the most widely consumed ready‐to‐eat meat products worldwide. However, their high content of saturated fatty acids and susceptibility to lipid oxidation pose significant nutritional and technological challenges, motivating the development of healthier burger formulations (Bamdad et al. 2025; Karimi Sani et al. 2024; Rengifo et al. 2024). Functional foods are defined as products that provide physiological benefits beyond basic nutrition, either through naturally occurring bioactive compounds or through deliberate formulation strategies. In this context, plant‐derived oils rich in polyunsaturated fatty acids (PUFAs) and natural antioxidants have gained considerable attention as functional ingredients in meat systems (Abedinia et al. 2025).

Purslane ( *Portulaca oleracea* L.) has been recognized as one of the richest plant sources of α‐linolenic acid among leafy vegetables, making it a promising functional lipid ingredient (Salehi et al. 2021). Purslane seed oil (PO) contains high levels of PUFAs (≈58%–68%) compared to saturated fatty acids (≈18%–21%), offering clear nutritional advantages over conventional animal fats (Delfan‐Hosseini et al. 2017; Petropoulos et al. 2020). In addition, purslane seeds are a valuable source of tocopherols and phenolic compounds with antioxidant activity (Mitroi et al. 2024). Despite these nutritional benefits, the high unsaturation degree of PO makes it highly prone to oxidative degradation during processing and storage, limiting its direct application in meat products.

Encapsulation technologies have therefore been proposed to enhance oxidative stability and improve technological performance of PUFA‐rich oils (Jurić et al. 2022). Spray drying is one of the most widely applied encapsulation techniques in the food industry due to its scalability, cost‐effectiveness, and ability to produce stable powders with low water activity, making it suitable for lipid‐based functional ingredients (Geranpour et al. 2020). Lipid oxidation remains a major cause of quality deterioration in meat products, leading to off‐flavors, discoloration, nutrient loss, and reduced consumer acceptance (dos Santos et al. 2024; Fathali Beygi et al. 2025; Khajeh et al. 2025). Natural antioxidants are therefore increasingly favored over synthetic additives due to their dual preservative and health‐promoting functions (Gorzin et al. 2024; 2026).

Tarragon ( *Artemisia dracunculus* L.) essential oil exhibits strong antioxidant and antimicrobial activities, largely attributed to its dominant phenylpropanoid components such as estragole, and has been proposed as a natural preservative in meat systems (Azizkhani et al. 2021; Khajeh et al. 2025). The co‐encapsulation of purslane seed oil and tarragon essential oil (PO–TE) represents a rational formulation strategy that combines the nutritional benefits of PUFA enrichment with the preservative efficacy of bioactive essential oils. This approach not only mitigates the oxidative instability of purslane oil but also enables synergistic antioxidant and antimicrobial effects while controlling release behavior (Azarashkan et al. 2022; Chasquibol et al. 2023; Farahani et al. 2025). Previous studies have shown that co‐encapsulation of oils and essential oils can improve bioavailability, physicochemical stability, and controlled release properties, facilitating their application in complex food matrices (İncili et al. 2025; Rehman et al. 2025).

GA and WPI demonstrate robust potential for simultaneously encapsulating seed and essential oils as natural preservatives in meat products, with (Mahdi et al. 2021) showing encapsulation efficiencies up to 92.08%. However, limited information is available on the simultaneous nano‐encapsulation of PUFA‐rich seed oils with essential oils for meat product reformulation, particularly regarding their combined effects on oxidative stability, microbial quality, sensory attributes, and fatty acid profile. Therefore, the present study aimed to address this research gap by investigating the co‐encapsulation of PO–TE using GA and WPI, and evaluating the functionality of the resulting nanocapsules as natural preservatives and functional lipid replacers in beef burger formulations.

## 2 Materials and Methods

### 2.1 Materials

Purslane seeds (*Portulaca oleracea* L.) were purchased from *grocery* stores of Shiraz (Iran). Tarragon was collected from the lands of Damghan Agricultural University (Iran). Whey protein isolate (> 95%, Batch No.: NPT19) and gum Arabic (CAS No.: 9000‐01‐5) were purchased from BiPro GmbH, (Germany) and Sigma Aldrich (Canada), respectively. All chemicals and culture media used in this research were purchased from Merck Co. (Germany).

### 2.2 Preparation of Purslane Oil (PO) and Determination of Its Fatty Acid Profile

A conventional solvent extraction method was used to extract oil from purslane seeds. In this method, 20 ± 0.01 g of purslane seeds were poured into a Soxhlet and the oil was extracted using n‐hexane solvent for a total extraction time of 6 h. The solution extracted by Soxhlet contained oil and hexane solvent, and the hexane solvent was separated under vacuum (temperature 65°C ± 0.1°C) using a rotary evaporator (Heidolph, Germany). Finally, minor amounts of the remaining solvent were removed using a gentle stream of nitrogen (Hossein Zadeh et al. 2023).

Gas chromatography (GC) (Agilent Technologies‐5975C‐MS, 7890A‐GC, America) was used to determine the fatty acid profile of PO. Initially, fatty acid methyl esters were prepared. For this purpose, PO (0.1 ± 0.001 g) was dissolved in n‐hexane (10 ± 0.01 mL) and centrifuged for 30 min at 4500 rpm. After that, 5.0 ± 0.01 mL methanolic potassium hydroxide (2 N) was added and stirred. The resulting mixture was kept at 25°C ± 1°C in the dark place for 30 min to allow phase separation. The supernatant (1 ± 0.001 mL) was transferred to a glass vial to be used for injection into GC. The GC system used was equipped with a flame ionization detector (FID) and a capillary column (length 30 m, width 0.32 mm, and particle size 0.25 μg). The temperature of the detector and injection section was set at 260°C. Nitrogen gas with a flow rate of 1.51 mL/min was used as the carrier gas. The oven temperature program was set in such a way that the temperature was initially maintained at 210°C for 9 min and then increased to 350°C at a rate of 20°C/min over 10 min. The sample was injected into the device in a volume of 1 ± 0.01 μL. To identify the fatty acid composition of the PO, the obtained retention times were compared with those of authenticated fatty acid methyl ester standards, and compound identification was confirmed using the NIST mass spectral library (version 14) and retention index matching (Al Juhaimi et al. 2025; Soleimani et al. 2025).

### 2.3 Extraction of Tarragon Essential Oil (TE) and Identification of Its Phytochemical Compounds

Clevenger method was used to prepare tarragon essential oil. The ratio of plant to distilled water was 1:5 and the extraction time was 4 h. The extracted essential oil was dried with anhydrous sodium sulfate and stored in dark glass vials at freezer temperature (−20°C ± 0.01°C) until further use. Identification of phytochemical compounds of extracted tarragon essential oil was performed by a gas chromatography–mass spectrometry (GC–MS) system equipped with a capillary column with dimensions of 30 m × 0.25 mm (film thickness 0.25 ± 0.01 μm) and a flame ionization detector (FID). The injection section temperature was 250°C and the carrier gas used was helium at a flow rate of 1 mL/min. The injection volume was 1 ± 0.001 μL. The temperature program was set in the range of 60°C–300°C (rate 3°C/min) (Mehaya et al. 2024). Following extraction, the obtained essential oil was separated from the aqueous phase and dried over anhydrous sodium sulfate (≥ 99%, Merck, Germany) to remove residual moisture. Approximately 2–3 ± 0.01 g of sodium sulfate was added per 100 mL of oil, and the mixture was gently agitated for 30 min at room temperature (25°C ± 1°C). The desiccant was then removed by filtration through Whatman No. 1 filter paper. The dried essential oil was stored in amber‐colored glass vials at 4°C until further analysis to prevent oxidative degradation and preserve volatile constituents.

### 2.4 Simultaneous Encapsulation of PO and TE

In this study, whey protein isolate (WPI) and gum Arabic (GA) were used for simultaneous encapsulation of PO and TE. The wall materials constituted 20% (w/w) and the combination of PO and TE (at a fixed ratio of 1:1 w/w) constituted 10% (w/w) of the dry matter of the emulsions. The remaining 70% comprised the aqueous phase, consisting of deionized water containing the emulsifier and stabilizing agents. The mixture was homogenized at 12,000 rpm for 3 min using a high‐speed homogenizer (Ultra‐Turrax, IKA, Germany), followed by ultrasonication (amplitude 60%, power 200 W, continuous mode, 25°C, 5 min) to achieve uniform droplet size distribution (Hasani et al. 2023). First, the wall materials were poured separately into distilled water and kept under constant stirring at 25°C ± 1°C for 12 h to hydrate. Different ratios of GA and WPI (including 1:1, 1:0, 0:1, 3:1 and 1:3 w/w) were used. After adding equal mixtures of PO and TE to the wall solutions, an ultrasonic homogenizer (SONOPULS HD‐4200, Bandelin Co., Germany) was used for 10 min (14,000 rpm) to obtain a nanoemulsion. Then, the formed nanoemulsions were dried by spray drying (DSD‐06; Dorsa Tech, Iran) using a 0.7 mm nozzle diameter, atomization pressure of 2 bar, and feed rate of 6 mL/min at an inlet temperature of 150°C ± 0.1°C and an outlet temperature of 80°C ± 0.1°C, with a dispersion flow rate of 6 mL/min and a drying air flow rate of 35 m^3^/h (Ferraz et al. 2022). FTIR spectroscopy was used to confirm nanocapsule composition and successful encapsulation through characteristic functional group interactions.

#### 2.4.1 Determination of Average Particle Size, Polydispersity Index and Zeta Potential

The particle size and polydispersity index (PDI) of the nanocapsules were measured using dynamic light diffraction (DLS) and by a zetasizer (SZ 100, Japan) at an angle of 90°. The zeta potential was also measured. Before the test, each capsule sample was diluted with distilled water in a ratio of 1:20 w/v and the measurement was carried out at room temperature (H. Zhang et al. 2020).

#### 2.4.2 Measurement of Oil Encapsulation Efficiency

To determine the oil encapsulation efficiency (EE) of the nanocapsules, their surface oil content was first determined. In this way, hexane solvent (15 ± 0.01 mL) was added to each of the nanocapsule powders (1.5 ± 0.01 g) and shaking was carried out for 2 min. After passing through Whatman filter paper (No. 1), it was washed three times with hexane (20 ± 0.01 mL). The organic phase was collected and the solvent was evaporated (temperature 60°C ± 0.1°C). The resulting extract indicates the amount of oil remaining on the surface of the nanocapsules. Finally, the percentage of oil EE of the nanocapsules was obtained through the following equation (*n* = 3 independent replicates) (Gorzin et al. 2024):

$$\mathrm{EE}\;\left(\%\right)=\frac{\text{Amount of initial oil}-\text{Amount of surface oil}}{\text{Amount of initial oil}}\times 100$$

#### 2.4.3 Measurement of Antioxidant Activity

The antioxidant activity of nanocapsules was investigated using DPPH, ABTS^+^, and Ferric reducing ability (FRAP) radical scavenging methods. To investigate antioxidant activity using the DPPH (2,2‐Diphenyl‐1‐picryl‐hydrazyl) method, 0.5 ± 0.1 mg of each sample was dissolved in 2 ± 0.001 mL of DPPH methanolic solution (0.004%) in a glass tube and stirred vigorously. The mixture was kept at 25°C ± 1°C and in the dark for 30 min, and then the absorbance of the samples was recorded at 517 nm. The solution without sample was used as a blank. The percentage of DPPH radical scavenging of the samples was obtained by the following equation (Gorzin et al. 2024):

$$\text{DPPH}\;\left(\%\right)=\frac{A\left(\text{blank}\right)-A\;\left(\text{sample}\right)}{A\left(\text{blank}\right)}\times 100$$

To investigate the antioxidant activity of nanocapsules by ABTS^+^ method, ABTS (7 mmol/L) and potassium persulfate (2.45 mmol/L) solutions were mixed in a 1:1 v/v ratio and the resulting mixture was kept in the dark at 25°C ± 0.01°C for 16 h, thus preparing ABTS radical solution. Then, ABTS radical solution was diluted with distilled water until an absorbance of ~0.7 (at a wavelength of 734 nm) was reached. Each of the samples was dissolved in distilled water in a ratio of 1:100 v/v, and then ABTS radical solution (280 ± 0.01 μL) was transferred to the microplate along with the sample (20 ± 0.01 μL). Two blank and control solutions of the sample were also prepared. To prepare the blank solution, diluted solvent (20 ± 0.01 μL) and ABTS radical solution (280 ± 0.01 μL) were used, and the control solution of each sample contained 20 ± 0.01 μL of sample and distilled water (280 ± 0.01 μL). The microplate was kept in the dark for 30 min at 25°C ± 0.01°C and its absorbance was recorded at 734 nm, and the results were expressed in terms of mg of Trolox equivalent per gram of dry weight (mg TE/g DW) (Silva et al. 2023).

To measure the antioxidant activity by the iron reducing power (FRAP) method, first acetate buffer (25 ± 0.01 mL), TPTZ solution (2,3,5‐Triphenyltetrazolium chloride) (2.5 ± 0.01 mL) and FeCl_3_.6H_2_O solution (2.5 ± 0.01 mL) were mixed together and the resulting mixture was placed in a water bath at 37°C ± 0.2°C for 30 min. The resulting solution is called FRAP solution. Next, the FRAP solution (810 ± 0.01 μL) was mixed with the sample (90 ± 0.01 μL) and kept in the dark at 25°C ± 0.01°C for 30 min to form the Ferrous tripyridyltriazine complex. The absorbance of the solution was then measured by spectrophotometer (UV‐550, Jasco, Japan) at 595 nm. The Trolox (TE) curve was used to determine the FRAP values (Sai‐Ut et al. 2023).

#### 2.4.4 Investigation of Surface Morphology

The surface morphology of nanocapsules was investigated using a scanning electron microscope (SEM) (Tesca‐Vega3, Tescan Co.; Czech Republic) at an accelerated voltage of 15 kV (Zahed et al. 2023).

#### 2.4.5 Investigation of FTIR Spectroscopy

To investigate the FTIR spectra of nanocapsules to determine the functional groups, first 100 ± 0.1 mg of KBr was added to 1 ± 0.0001 g of each nanocapsule and compression was performed to convert them into tablets. The FTIR spectra were investigated using an infrared spectrometer (IR Affinity, Shimadzu, Japan) at room temperature and in the range of 700–4000 cm^−1^ and at a resolution of 4 cm^−1^ (Lin et al. 2019).

#### 2.4.6 Investigation of Thermal Stability

To investigate the thermal stability of nanocapsules, the thermogravimetric analysis (TGA) (SDT 650; TA Instruments, USA) method was used. The sample weight used in this method was 5 ± 0.1 mg and the analysis was performed in the temperature range between 25°C and 300°C with a heating rate of 10°C/min. To ensure controlled conditions during the experiment, nitrogen was used at a constant flow rate of 20 mL/min (Zhou et al. 2022).

### 2.5 Preparation of Burger Treatments

The beef burger formulation was as follows: ground beef, thigh and gizzard marrow (60.0% ± 0.5% w/w), onion (30.0% ± 0.3% w/w), breadcrumbs (8.0% ± 0.1% w/w), salt and spices including 1.7% ± 0.05% sodium chloride (NaCl) and a standardized spice mixture (0.3% ± 0.02%). All ingredients were thoroughly mixed to achieve a homogeneous texture before shaping into patties. First, the meat and fat were ground using a meat grinder, and then all the formulation components were mixed well together until a uniform dough was obtained. The dough was molded and stored at refrigerator temperature (4°C ± 0.5°C) for 12 days and tested every 3 days (Mehaya et al. 2024). Nanocapsules were added to the burger formulation at levels of 0%, 0.9%, 1.8% and 2.7% (C, N0.9, N1.8 and N2.7) and replaced part of the meat fat. The total mass of each burger patty was 100 g ± 1 g, molded using a standardized circular mold (diameter 9 cm, thickness 1.5 cm). Burgers were cooked in triplicate (*n* = 3) per treatment.

#### 2.5.1 pH Measurement

The pH of the burgers was measured at room temperature using a pH‐meter (HM‐20S, Japan) calibrated with buffers of pH 4 and 7. For this purpose, each of the samples (10 ± 0.01 g) was homogenized in distilled water (10 ± 0.01 mL) (1 min at 6000 rpm) and then their pH was recorded (Cao et al. 2024).

#### 2.5.2 Measurement of Total Volatile Basic of Nitrogen (TVB‐N)

A 10 ± 0.01 g of burger, 2 ± 0.01 g of magnesium oxide along with 300 ± 0.01 mL of distilled water were transferred into a Kjeldahl flask and the flask was heated from below. At the end of the Kjeldahl system, an Erlenmeyer flask (250 ± 0.1 mL) containing 25 ± 0.01 mL of 2% (w/v) boric acid solution and a few drops of methyl red reagent was added. The distillation process continued for about 45 min from the time the materials in the flask boiled and about 100 ± 0.1 mL of liquid was collected. The titration process was continued until a red color appeared with 0.1 N sulfuric acid. The TVB‐N content of burgers was reported in mg N/100 g (Socaciu et al. 2021).

#### 2.5.3 Peroxide Index Measurement

The peroxide index of burger samples was measured according to the method described by the AOCS standard (1998). Each sample (40 ± 0.01 g) was mixed with chloroform (100 ± 0.1 mL) and after passing through Whatman No. 44 filter paper, its solvent was evaporated using a rotary evaporator. To 5 ± 0.01 g of extracted fat, 37 ± 0.01 mL of a mixed solution of acetic acid‐chloroform (2:3 ratio v/v) and then saturated potassium iodide solution (1 ± 0.001 mL) were added and after 1 min, distilled water (30 ± 0.01 mL) and starch solution (1 ± 0.001 mL) were added. The mixture was titrated until the yellow color disappeared using 0.01 N sodium thiosulfate solution. The peroxide index of burgers was obtained using the following equation (Ashrafi et al. 2023):

$\mathrm{POV}\;\left(\mathrm{meq}/\mathrm{kg}\right)=\frac{0.1\times \text{Volume of thiosulfate}}{\text{Weight of sample}}\times 100$.

#### 2.5.4 Measurement of Thiobarbituric Acid (TBA) Index

To determine the TBA index of burgers, first a 1% TBA solution was prepared by mixing TBA (5 ± 0.01 g), sodium hydroxide (1.5 ± 0.01 g) and distilled water (500 ± 0.1 mL) and a trichloroacetic acid solution was prepared by mixing trichloroacetic acid (12.5 ± 0.01 g), 0.6 M hydrochloric acid (3 ± 0.01 mL) and distilled water (500 ± 0.1 mL). Then, in a centrifuge tube (50 ± 0.1 mL), 2 ± 0.001 g of each sample was poured along with 3 ± 0.001 mL of TBA solution and 17 ± 0.01 mL of 1% w/v trichloroacetic acid solution and mixed together. The resulting mixture was heated for 30 min at 100°C ± 2°C and after cooling to room temperature, the supernatant (4 ± 0.01 mL) was mixed with an equal volume of chloroform and centrifuged for 10 min at 3000 rpm. The absorbance of each sample solution was recorded at 532 nm and finally, the TBA index of the burgers was obtained in mg MDA/kg (mg of malondialdehyde per kg of sample) using the following equation (Cao et al. 2024):

$$\mathrm{TBA}\;\left(\mathrm{mg}\;\mathrm{MDA}/\mathrm{kg}\right)=9.48\times \left(\frac{\text{Absorbance}}{\text{Weight of sample}}\right)$$

#### 2.5.5 Determining Protein Oxidation

The oxidation of proteins was studied using total carbonyl content determination. First, 2.5 ± 0.001 g of burger was homogenized with urea solution (8 M) (20 s) and then, from it, 10% w/v trichloroacetic acid solution (1 ± 0.01 mL) was extracted to extract the sample protein. The mixture was centrifuged for 5 min at 5000 × g and then 2 M hydrochloric acid (1 ± 0.01 mL) and hydrochloric acid solution containing 0.2% w/v dinitrophenylhydrazine (100 ± 0.01 μL) were added. After that, dissolution was carried out in 20 mM sodium phosphate buffer solution (1.5 ± 0.01 mL) containing guanidine hydrochloric acid (M 6; pH = 6.5) and mixed well. The mixture was centrifuged for 2 min at 4200 × g and its absorbance was recorded at 280 nm. The bovine serum albumin standard curve was used to calculate the amount of burger carbonyl and the carbonyl content of burgers was reported in nmol/mg protein (Ashrafi et al. 2024).

#### 2.5.6 Measurement of Cooking Loss

The burgers were cooked in an electric oven at 180°C ± 5°C until the temperature of the center of the burgers reached 80°C ± 2°C and the percentage of cooking loss of the samples was obtained using the following equation (Shoqairan et al. 2023):

$$\text{Cooking loss}\;\left(\%\right)=\frac{\text{Weight before cooking}-\text{Weight after cooking}}{\text{Weight before cooking}}\times 100$$

#### 2.5.7 Determination of Free Fatty Acid Profile

The fatty acid profile of burger samples was determined according to the method described for determining the fatty acid profile of PO and using the GC system (Al Juhaimi et al. 2025).

#### 2.5.8 Color Examination

The color of the samples was examined using the calorimetric method (Minolta, China) and the color indices L*, a* and b*, which indicate the intensity of light, red‐green, and yellow‐blue color, respectively, were determined. Before performing the analysis, the device was calibrated with a white ceramic plate (Foggiaro et al. 2022).

#### 2.5.9 Examination of Textural Parameters

Measurement of textural parameters including firmness (N), cohesiveness, chewiness (N mm), springiness (mm) was carried out at room temperature using a texture analyzer (Instron, model 6025, England) and a cylindrical probe with a flat surface. Compression was performed to 60% of the initial height of the samples, and the crosshead speed was set at 3.33 mm/s (Foggiaro et al. 2022).

#### 2.5.10 Microbial Load Analysis

To count the total viable mesophilic bacteria (TVMBC) of burgers, first the desired dilution was prepared from each sample. For this purpose, 20 ± 0.01 g of each sample was transferred to Stomaker bags containing 0.9% w/v sterile peptone water (180 ± 0.01 mL) and homogenization was performed at 230 rpm for 45 s. After homogenization, serial decimal dilutions (10^−1^ to 10^−7^) of each burger sample were prepared in sterile 0.85% w/v saline solution. The selection of this dilution gradient was based on preliminary testing to ensure that the resulting plate counts fell within the standard countable range of 30–300 CFU per plate, thereby providing statistical reliability and minimizing sampling error. The linear range of the plate counting confirmed that colony counts within this range exhibit a direct proportional relationship with microbial concentration. Only plates with colonies within this range were used for calculating the final microbial load, expressed as log CFU/g of sample. To count the total viable bacteria, each of the dilutions prepared from burger samples (0.1 ± 0.001 mL) was surface‐cultured on Plate Count Agar (PCA) medium and then incubated at 30°C ± 0.2°C for 2 days. Finally, the number of colonies formed was counted and reported based on the logarithm of the number of colonies per gram of sample (log CFU/g) (Ebrahimi et al. 2022).

#### 2.5.11 Sensory Evaluation

The sensory characteristics flavor (salty, umami, meaty, and off‐flavor notes), color (intensity and uniformity), odor (fresh and rancid attributes), texture (tenderness and juiciness), and overall acceptability of cooked burgers were evaluated using a 7‐point hedonic scale (1 = dislike extremely, 2 = dislike moderately, 3 = dislike slightly, 4 = neither like nor dislike, 5 = like slightly, 6 = like moderately, and 7 = like extremely) by 20 panelists (10 men and 10 women). Cooked burgers were provided to the raters in coded white plastic containers (randomly) along with water and a sensory evaluation form, and the raters were given basic explanations about how to score and evaluate the characteristics (Shahabi et al. 2024). The evaluation was performed in a sensory laboratory under controlled conditions (22°C ± 2°C, neutral white lighting, and between 10:00 a.m. and 12:00 p.m.) to minimize bias due to environmental factors. All participants were untrained volunteers who were regular consumers of meat products and received standard instructions before the test. The study protocol was approved by the institutional ethics committee that conformed to the Declaration of Helsinki, and informed consent was obtained from all participants prior to the sensory evaluation.

### 2.6 Statistical Analysis of Data

Data normality was assessed using the Shapiro–Wilk test, and no data transformation was required. One‐way and two‐way ANOVA models were applied depending on the variable structure. Statistical analyses were performed using SPSS software version 22.0 (IBM Corp., USA). Duncan's multiple range test was used to express statistically significant differences between samples at the 95% probability level (*p* < 0.05). Finally, all experimental results were reported as mean ± standard deviation (*n* = 3 independent replicates per treatment), ensuring analytical robustness and reproducibility.

## 3 Results and Discussion

This section not only reports the experimental outcomes but also interprets the observed differences between encapsulation systems in terms of biopolymer composition, interfacial behavior, and interactions with bioactive compounds, aiming to provide formulation‐relevant insights for functional food applications.

### 3.1 Phytochemical Compounds of TE

The TE phytochemical compound used in this study was identified by GC–MS system and the type and amount of these compounds are given in Table 1. Overall, 14 phytochemicals were found in TE, with methylcavicol or estragole (83.71%) being the most dominant compound, followed by (Z)‐β‐ocimene (19.5%), trans‐ocimene (3.47%), limonene (3.24%), and β‐pinene (1.15%), respectively. Other compounds were present in minor amounts. In a study by Sahakyan et al. (2021), estragole (84.9%), linalool (5.09%), β‐ocimene (4.00%), (Z‐E)‐allocymene (2.29%), and limonene (1.63%) were the major phytochemicals in TE. In another study, estragole (79.42%), cis‐β‐ocimene (8.42%), trans‐β‐ocimene (6.69%), limonene (3.13%), and α‐pinene (1.00%) were reported as the main phytochemical compounds of TE (Pujicic et al. 2025).

**TABLE 1:** Phytochemical composition of tarragon essential oil (TE).

No	Retention time	Compounds	Values (%)
1	11.15	α‐pinene	1.15
2	13.36	β‐pinene	0.08
3	14.21	β‐myrcene	0.03
4	16.02	Limonene	3.24
5	16.51	(Z)‐β‐ocimene	5.19
6	17.29	Trans‐ocimene	3.47
7	18.96	Terpinene	0.11
8	19.71	Linalool	0.65
9	21.19	Allo‐ocymene	0.1
10	25.41	Estragole	83.71
11	28.03	Geranial	0.52
12	31.71	Eugenol	0.07
13	40.29	Cinnamaldehyde	0.35
14	40.89	Spanthulenol	0.07
		Total	98.74

In the essential‐oil profile of Tarragon (TE), estragole (CAS 140‐67‐0) is identified as a major constituent. The presence of estragole in TE products provides antimicrobial and antioxidant effects but animal research indicates this compound has genotoxic and carcinogenic effects, which creates uncertainty about safe human exposure levels. The European Food Safety Authority (EFSA), along with other regulatory organizations, have determined that current scientific evidence does not support any particular human exposure limit for estragole (Tata et al. 2025).

Nanoemulsion and co‐encapsulation techniques reduce estragole volatility in food application systems. Controlled‐release systems and dose reduction produce continuous antimicrobial compound release. The active‐film system combines nanoemulsified TE with biopolymeric nanoparticles for enhanced antimicrobial effects. Full‐scale studies are needed to establish sustainable operations and determine bioaccessible levels. Future research should measure residual estragole content and test bioaccessibility after entering the human digestive system.

### 3.2 Fatty Acid Profile of PO

The fatty acid profile of PO used in this study was determined by the GC system and the type and percentage of fatty acids present in the oil of this seed are shown in Table 2. The dominant fatty acid of this oil was oleic acid (43.19%), followed by α‐linolenic fatty acids (26.42%), palmitic acid (13.33%), and oleic acid (11.37%), respectively. The largest share of PO fatty acids was made up of polyunsaturated fatty acids (PUFA) (69.61%), followed by saturated fatty acids (SFA) (17.91%), and monounsaturated fatty acids (MUFA) (12.48%). It has been stated that if the ratio of PUFA to SFA is less than 4 and the n6/n3 ratio is higher than 0.45, the oil in question has high nutritional value (Petropoulos et al. 2021). Accordingly, due to the high nutritional value of PO, its use in the human diet is recommended. In the study conducted by Hossein Zadeh et al. (2023), linoleic acid (32.36%), linolenic acid (29.38%), oleic acid (17.80%), and palmitic acid (14.89%) were reported as PO fatty acids, respectively. In another study, linolenic acid (39.57%), linoleic acid (35.13%), palmitic acid (14.18%), and oleic acid (5.78%) were reported as the dominant PO fatty acids, respectively. These researchers also stated, in agreement with the results of the present study, that the largest contribution of PO fatty acids was related to polyunsaturated, saturated, and monounsaturated fatty acids, respectively (Petropoulos et al. 2020). Linolenic fatty acid is known as omega‐3, linoleic fatty acid as omega‐6, and oleic fatty acid as omega‐9. These free fatty acids are in the category of essential fatty acids for the body. The differences in the fatty acid content of PO reported by different researchers can be attributed to differences in geographical region, genotype, growing conditions, method, and conditions of oil extraction (Delfan‐Hosseini et al. 2017).

**TABLE 2:** Fatty acid composition of purslane oil (PO).

Fatty acids	Composition (%)
Lauric acid (C12:0)	0.21 ± 0.05
Myristic acid (C14:0)	0.50 ± 0.02
Palmitic acid (C16:0)	13.33 ± 0.01
Palmitoleic acid (C16:1)	0.86 ± 0.02
Stearic acid (C18:0)	3.24 ± 0.08
Oleic acid (C18:1n9)	11.37 ± 0.04
Linoleic acid (C18:2n6)	43.19 ± 0.04
α‐Linolenic acid (C18:3n3)	26.42 ± 0.05
Behenic acid (C22:0)	0.44 ± 0.02
Erucic acid (C22:1)	0.25 ± 0.01
Lignoceric acid (C24:0)	0.19 ± 0.04
SFA	17.91 ± 0.10
MUFA	12.48 ± 0.08
PUFA	69.61 ± 0.05
PUFA/SFA	3.89 ± 0.06
n6/n3	1.63 ± 0.02

### 3.3 Characteristics of PO‐TE Nanocapsules

#### 3.3.1 Average Particle Size, PDI and Zeta Potential

Average particle size and PDI index are important and key factors that play a significant role in evaluating the efficiency of encapsulation, the release rate of the core active compounds and the stability of emulsions (Habib et al. 2025). The results (Table 3) showed that the nanocapsule prepared by GA coating alone had a significantly smaller particle size (293.7 nm) than the WPI coating alone (293.7 nm). The use of a combination of GA and WPI led to the production of capsules with a larger size and with increasing the WPI ratio in the coating, the particle size of the capsules also increased, so that the largest average particle size was for the nanocapsule prepared with GA1:WPI3 (665.8 nm). The production of smaller particles by GA is probably related to the favorable emulsifying property of this biopolymer. Because GA has peptide chains in its structure that can be connected to oil droplets and other emulsion components through covalent bonds and form smaller particles (Shahidi Noghabi and Molaveisi 2020). The use of a combination of wall materials resulted in the production of larger capsules compared to the single use of biopolymers, which is probably related to the increase in the thickness of the wall layer around the oil droplets and can also show better ability to preserve active compounds (Habib et al. 2025). In terms of PDI, the lowest value was related to the coatings GA1:WPI0 (0.384) and GA3:WPI1 (0.392), and there was no statistically significant difference between these two samples. The nanocapsule prepared with GA1:WPI3 coating (0.523) had the highest PDI, and with the exception of this sample, the other samples had a PDI index of less than 0.5 and had a suitable particle size distribution. Chasquibol et al. (2023) also showed in their study that the size of the capsule prepared by GA alone was smaller than that prepared by the GA‐maltodextrin‐WPI combination. In the study conducted by Al‐Maqtari et al. (2021), the of *Pulicaria jaubertii* extract microencapsulated in an equal combination of whey protein and GA were reported to be 465.87 and 0.30 nm, respectively, which the size of the nanocapsules produced by them was larger than that of the present study.

**TABLE 3:** Mean particle size, PDI, zeta potential and EE of PO–TE nanocapsules.

Samples	Z‐average (nm)	PDI	Zeta potential (mV)	EE (%)
GA0: WPI1	585.4 ± 9.7^c^	0.464 ± 0.013^b^	−27.5 ± 0.8^b^	78.34 ± 0.75^e^
GA1: WPI0	293.7 ± 13.1^e^	0.384 ± 0.018^d^	−28.3 ± 1.5^b^	83.19 ± 1.02^d^
GA3: WPI1	489.8 ± 15.5^d^	0.392 ± 0.009^d^	−33.0 ± 2.4^a^	90.79 ± 0.71^a^
GA1: WPI1	635.3 ± 10.2^b^	0.425 ± 0.015^c^	−29.3 ± 1.9^ab^	88.65 ± 0.93^b^
GA1: WPI3	665.8 ± 16.9^a^	0.523 ± 0.010^a^	−26.5 ± 2.1^b^	85.03 ± 0.66^c^

*Note:* Values are expressed as mean (*n* = 3) ± standard deviation. Different lowercase letters within a row indicate significant differences among samples (*p* < 0.05), while different uppercase letters indicate significant differences across storage periods (*p* < 0.05).

Abbreviations: PDI, polydispersity index; PO–TE, purslane oil–tarragon essential oil; WPI, whey protein isolate.

Zeta potential has a strong relationship with the stability of particles and emulsions in colloidal systems. So that if its value is greater than ±25 mV, the emulsion will have good stability (Al‐Maqtari et al. 2021). In general, van der Waals forces between particles are responsible for their aggregation, and increasing the zeta potential values of particles, by reducing these forces, leads to a decrease in the tendency to aggregate and an increase in the stability of particles (Jhan et al. 2020). Stable particles are suitable for medical and food applications. All nanocapsules produced in this study had a negative surface charge (Table 3). With the exception of the nanocapsule prepared with the GA3:WPI1 coating, which had the highest zeta potential (−0.33 mV), no statistically significant difference was observed between the zeta potential values of other nanocapsules, and their zeta potential values were in the range of −26.5 to −29.3 mV. All nanocapsules show favorable stability in colloidal systems due to their zeta potential higher than −25 mV. Rashid et al. (2022) observed that the zeta potential of pomegranate peel extract nanocapsules produced by a combined coating of polysaccharide (maltodextrin) and protein (WPI) was higher than that of single coatings. Shao et al. (2019) also achieved similar results during the encapsulation process of resveratrol by different coatings. These researchers also reported the negative charge and high zeta potential of capsules prepared by GA and whey protein and observed that the zeta potential increased with increasing the percentage of GA in the wall material used. Habib et al. (2025) also showed that the zeta potential of astaxanthin nanocapsules prepared by a combined coating of GA‐WPI was higher than that of single coatings.

The smaller particle size observed in gum arabic–coated nanocapsules can be mechanistically attributed to its highly branched arabinogalactan–protein structure, which enhances interfacial adsorption and rapid stabilization of oil droplets during emulsification (Gorzin et al. 2024). In contrast, whey protein isolate–rich systems tend to form thicker interfacial layers due to protein unfolding and aggregation, leading to increased particle size but potentially improved barrier properties against oxygen diffusion (Habib et al. 2025).

#### 3.3.2 EE

The EE test was used to evaluate the efficiency of the coating process and the coatings used to trap the oil inside the capsules, so that the higher the EE percentage, the better the system used in preserving the central active compounds. The EE values of PO‐TE nanocapsules prepared by different coatings are compared with each other in Table 3 and show that the combined coatings had higher EE compared to the single coatings (*p* < 0.05) and the EE of the nanocapsule prepared with GA alone (83.19%) was higher than the WPI coating (78.34%). With increasing the ratio of GA to WPI in the coating of the nanocapsules, the EE percentage increased significantly (*p* < 0.05), so that the highest EE was for the nanocapsule prepared with GA3:WPI1 (90.79%). Milk proteins also show good ability to retain the core active compound, as they are amphiphilic in nature and exhibit biological activity, emulsifying properties, high coating and film‐forming ability (Akbarbaglu et al. 2021). Between GA and WPI alone, the carbohydrate coating showed a higher EE, which is probably related to the smaller size of nanocapsules prepared by GA compared to WPI, since according to research, the leakage of the core material from capsules with smaller particle size is less and hence capsules with smaller size often have higher microencapsulation efficiency and show better ability to retain the core active agent (Premi and Sharma 2017; Soleimani et al. 2025). The good ability of GA to create capsules with higher EE was also confirmed by Rezvankhah et al. (2022). Rehman et al. (2025) also showed that the use of a combined coating of protein isolate and gum showed higher EE of the oil compared to their single use. In the study of Al‐Maqtari et al. (2021), the EE of extract nanocapsules prepared by the WPI‐GA combination was reported to be 89.67%, which was lower than the value obtained in the present study. Karrar et al. (2021) found that the EE of capsules produced by carbohydrate coating was higher than that of protein coating during the encapsulation process of Gurum seed oil by different coatings. Shao et al. (2019) also showed that the lowest EE was related to resveratrol capsules prepared by whey protein alone and its combination with GA significantly improved the EE of the produced capsules. The higher encapsulation efficiency observed in GA–WPI combined systems can be explained by synergistic polysaccharide–protein interactions, where electrostatic attraction and hydrogen bonding enhance matrix cohesion and reduce diffusion pathways for oil leakage. Such hybrid interfacial structures are particularly advantageous in retaining volatile and oxidation‐sensitive compounds, making them suitable for functional ingredient delivery (Rezvankhah et al. 2022).

#### 3.3.3 Antioxidant Activity

The antioxidant activity of PO‐TE nanocapsules was investigated by three methods: DPPH radical scavenging, FRAP and ABTS^+^. The DPPH, FRAP and ABTS^+^ values of PO‐TE nanocapsules prepared by different coatings are compared with each other in Table 4 and indicate that the combined coatings had higher antioxidant activity compared to single coatings (*p* < 0.05) and the antioxidant activity of nanocapsules prepared with GA alone was higher than that of WPI coating in all three analysis methods. The DPPH, FRAP and ABTS values of PO‐TE nanocapsules were obtained in the range of 73.60%–86.03%, 3.82–5.23 mM TE and 14.19–18.17 mg TE/g, respectively. In terms of DPPH value, the highest value was related to the GA3:WPI1 sample. In terms of FRAP, GA3:WPI1 and GA1:WPI1 samples had the highest levels and there was no statistically significant difference between these two samples. In terms of ABTS, the highest activity was related to the GA1:WPI1 sample. Rehman et al. (2025) also showed that the encapsulation process of peppermint oil and borage seed oil mixture with protein isolate and gum, especially their combination, improved the antioxidant activity of the oils. Papoutsis et al. (2018) also stated that the use of polysaccharide‐protein complexes for encapsulation of active agents can improve the preservation of the core compounds compared to the single use of these coatings and attributed this to the reaction between these two types of biopolymers. Higher antioxidant activity of mango peel extract nanocapsules prepared by combining WPI and maltodextrin compared to their single use was also reported by Saborirad et al. (2024). The enhanced antioxidant activity in combined GA–WPI nanocapsules is not solely related to higher phenolic retention, but also to the protective microenvironment created by the biopolymer network, which limits oxygen accessibility and delays oxidative degradation of both phenolic compounds and unsaturated fatty acids (Chen et al. 2023).

**TABLE 4:** Antioxidant activity of PO‐TE nanocapsules.

Samples	DPPH (%)	FRAP (mM TE)	ABTS^+^ (mg TE/g)
GA0: WPI1	73.60 ± 0.86^e^	3.82 ± 0.14^c^	14.19 ± 0.53^d^
GA1: WPI0	77.85 ± 0.53^d^	4.10 ± 0.18^c^	15.78 ± 0.37^c^
GA3: WPI1	86.03 ± 0.70^a^	5.23 ± 0.21^a^	16.62 ± 0.19^b^
GA1: WPI1	83.54 ± 0.69^b^	5.15 ± 0.16^a^	18.17 ± 0.46^a^
GA1: WPI3	80.21 ± 0.58^c^	4.69 ± 0.25^b^	15.91 ± 0.33^c^

*Note:* Values are expressed as mean (*n* = 3) ± standard deviation. Different lowercase letters within a row indicate significant differences among samples (*p* < 0.05), while different uppercase letters indicate significant differences across storage periods (*p* < 0.05).

Abbreviations: PO–TE, purslane oil–tarragon essential oil; WPI, whey protein isolate.

#### 3.3.4 Surface Morphology

**FIGURE 1:**
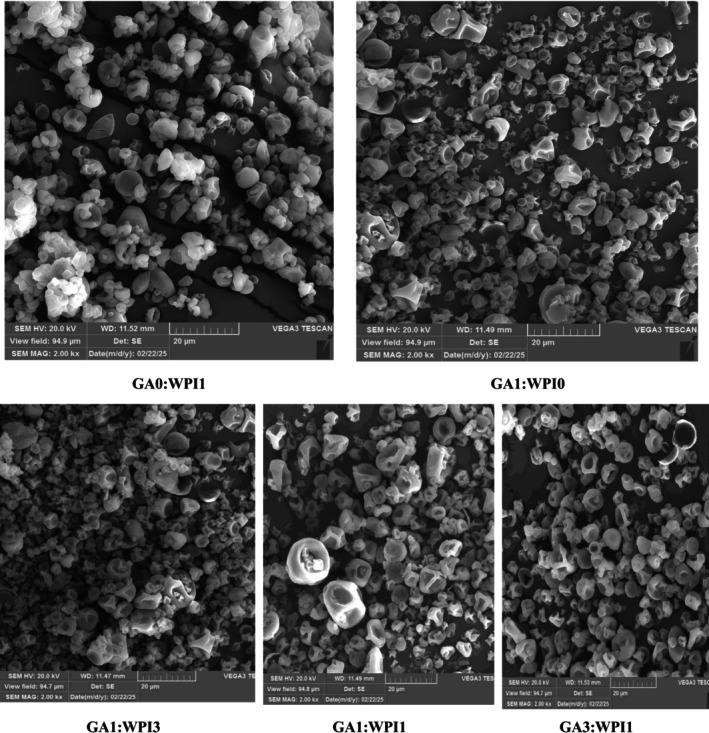
SEM images of PO‐TE nanocapsules. Magnification: 2000×; Scale bar: 20 μm. PO–TE = Purslane Seed Oil–Tarragon Essential Oil and wall materials (WPI and GA).

The surface morphology of PO‐TE nanocapsules prepared by different coatings was examined using SEM microscopy and the resulting images are presented in Figure 1. The nanocapsules produced in this study had an almost spherical shape with rough surfaces and surface depressions and had different sizes. This microstructure of the nanocapsules is related to their drying method, because during the spray drying process, spherical particles are formed that have a wrinkled surface and surface pores due to the use of high heat and rapid evaporation of water (Chen et al. 2023; Gan et al. 2024). Different sizes of particles produced by spray drying have also been reported by previous researchers (Chew et al. 2018). However, no cracks were observed on the surface of the produced nanocapsules, indicating their good stability and the creation of their desired microencapsulation efficiency. In general, the spherical shape of the capsules without cracks can protect the encapsulated oil from migration to the surface, exposure to oxygen and ultimately oxidation (Chasquibol et al. 2023). These findings were also reported in the study conducted by Karrar et al. (2021) on the microencapsulation of gurum seed oil with different wall materials (GA, maltodextrin and WPI). Due to the zeta potential of the nanocapsule prepared with WPI coating alone, a little adhesion was observed in the SEM image of this nanocapsule, while in the other nanocapsules favorable stability was observed and no aggregation was found.

#### 3.3.5 FTIR

**FIGURE 2:**
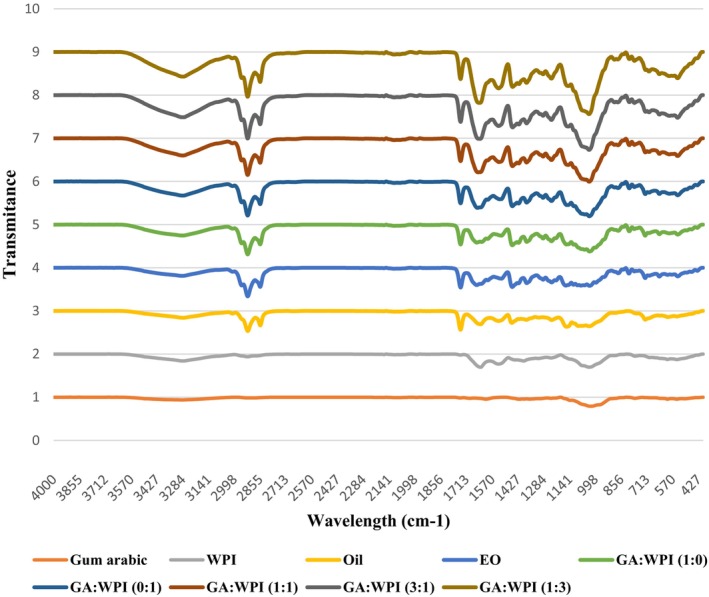
FTIR spectra of PO, TE, PO–TE nanocapsules and wall materials (WPI and GA) showing key functional groups and wavenumber assignments (4000–500 cm^−1^). FTIR, Fourier Transform Infrared Spectroscopy; PO–TE, Purslane Seed Oil–Tarragon Essential Oil: PO.

To investigate the functional groups of PO‐TE nanocapsules and raw materials (PO, TE and wall materials), FTIR spectroscopy was used and the resulting spectra are presented in Figure 2. In the FTIR spectrum of GA gum, weak peaks were observed in the regions of 3328 and 1614 cm^−1^, which were related to the O‐H stretching and N‐H bending or C=O stretching bands, respectively. In this spectrum, there was a larger and more significant peak in the region of 1016 cm^−1^, which was related to the C‐O stretching band. In the FTIR spectrum of WPI, peaks were also observed in the regions of 3285 cm^−1^ (O‐H stretching), 1628 cm^−1^ (amide type I), 1524 cm^−1^ (amide type II), 1391 cm^−1^ (C‐N stretching) and 1013 cm^−1^ (C‐O‐H bending and C‐O stretching). These peaks were also reported in the study of Karrar et al. (2021). In the spectra of PO and TE, similar peaks were observed and these peaks were observed in the regions of 3307 cm^−1^ (O‐H stretching vibrations), 2924 and 2847 cm^−1^ (C‐H stretching vibrations), 1738 and 1627 cm^−1^ (C=C stretching vibrations), 1141 (C‐O‐C stretching vibrations) and 1023 cm^−1^ (C‐O bending) (Kasiri and Fathi 2018). Examination of the FTIR spectra of PO‐TE nanocapsules prepared with different coatings showed that all the peaks present in the spectra of oil and essential oil were present in the spectra of nanocapsules, indicating the placement of these active agents in the coatings of interest. However, most of these peaks were more intense in the spectra of nanocapsules, indicating the establishment of bonds, especially hydrogen bonds (enlargement of the peak in the 3300–3200 cm^−1^ region) between the core active materials and the wall materials. In agreement with the results of microencapsulation efficiency and antioxidant activity, nanocapsules prepared with the combination of GA and WPI had higher phenolic compounds content than the single coatings, because their hydroxyl group peaks were larger. The establishment of hydrogen bonding between peppermint oil and the nanocapsule wall material was also observed by Karrar et al. (2021) and Gan et al. (2025). These results were also consistent with the findings of Rehman et al. (2025). An increase in the intensity of the hydroxyl group peak in hemp seed oil capsules prepared by the combination of GA, maltodextrin and WPC coatings compared to the crude oil was also reported in the study of Rezvankhah et al. (2022).

#### 3.3.6 Thermal Stability

**FIGURE 3:**
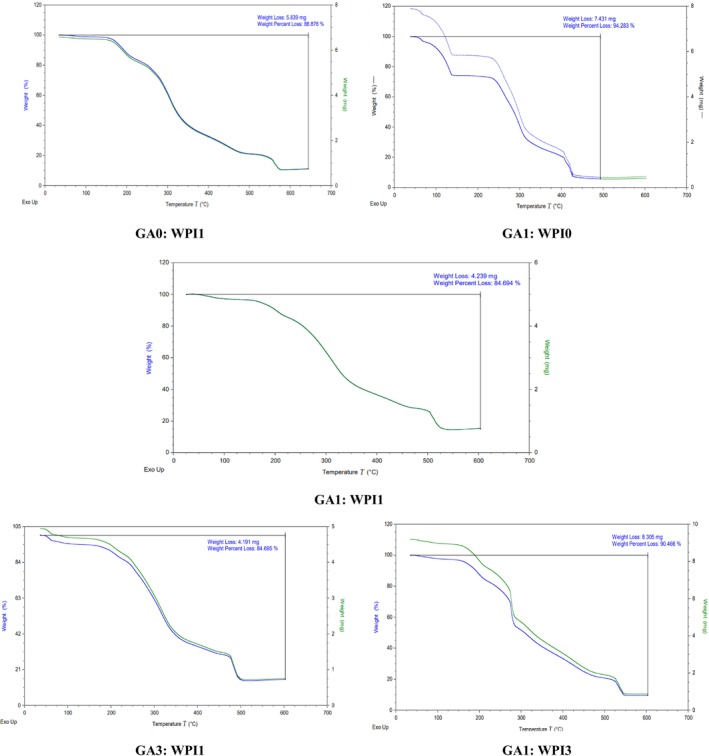
TGA thermograms of PO–TE nanocapsules and wall materials (WPI and GA) at temperature range between 25°C and 300°C with a heating rate of 10°C/min.

The thermal stability of PO‐TE nanocapsules prepared by different coatings was investigated using the thermogravimetric method (TGA) and the resulting curves are shown in Figure 3. As can be seen in the figure, the weight loss of the nanocapsules increased with increasing temperature. All samples showed several stages of weight loss. The first weight loss occurred at a temperature between 150°C and 160°C, which is related to the evaporation of excess water. Weight loss also occurred between this temperature and 225°C, which is related to the beginning of capsule destruction and separation of molecular chain connections. The highest percentage of weight loss was related to the temperature of 248°C to the range of 409°C–559°C. In the temperature range between 60°C and 500°C, the highest percentage of weight loss was observed in the nanocapsule prepared with the GA1:WPI0 coating, which was 94.28%, followed by the nanocapsules prepared with the ratios GA1:WPI3 (90.47%) and GA0:WPI1 (88.88%), respectively. The lowest percentage of weight loss was observed in the nanocapsules prepared with the ratios GA1:WPI1 (84.69%) and GA3:WPI1 (84.70%), and these two nanocapsules showed the highest thermal stability. The GA1:WPI0 formulation showed high weight loss (94.28%) and high encapsulation efficiency (EE = 83.19%), despite a significant amount of less‐volatile oil remaining encapsulated within the gum arabic matrix. This is consistent with previous reports suggesting that matrices rich in polysaccharides can retain core materials despite high evaporative loss during drying. The discrepancy may be due to differences in analytical endpoints, as TGA measures all volatile losses, while EE quantifies only oil retained versus surface oil. Microstructural factors like film porosity and thermal stability may promote moisture release but preserve internal oil entrapment. These clarifications help better understand the encapsulation behavior of the GA1:WPI0 formulation. The lower decomposition temperature of GA compared to WPI can be related to the presence of more chemical bonds in the WPI structure, because this protein isolate has hydrophobic forces and peptide bonds (Falsafi et al. 2022). The higher thermal stability in nanocapsules prepared by combining two biopolymers can be attributed to the formation of intermolecular forces between them and the central oil, and in any sample where these connections are stronger, the thermal stability is higher. Zhang et al. (2022) in their study addressed the microencapsulation of Spirulina chlorophyll by combined coatings of WPI and GA and, in agreement with the results of the present study, stated that the weight loss in the alone GA coating occurred at lower temperatures compared to WPI and its weight loss percentage was also higher. However, these researchers also observed that the weight loss percentage was lower in capsules prepared with a combined coating with an equal or higher proportion of GA. Tavares et al. (2019) showed that thermal degradation of capsules occurred in the temperature range of 150°C–400°C, and in this temperature range, the carbohydrate rings of the coatings used were degraded and the O‐O, S‐S, and O‐N bonds were destroyed. From a formulation perspective, the improved thermal stability of GA–WPI nanocapsules suggests their suitability for thermally processed meat products, where resistance to heat‐induced degradation is critical for maintaining bioactive functionality (Gorzin et al. 2024).

### 3.4 Characteristics of Burgers

#### 3.4.1 pH

The pH values of different burger treatments enriched with different levels of purslane oil‐tarragon essential oil nanocapsules are shown in Table 5. The combination of different levels of PO‐TE nanocapsules initially did not show a significant effect on the pH of the burgers. Over time, the pH of the burgers increased (*p* < 0.05), so that its level was in the range of 5.82–5.85 at the beginning of the storage period and reached 5.99–6.21 on the twelfth day. The increase in pH of the burgers during the storage period is related to biochemical changes caused by protein degradation and the production of volatile alkaline compounds such as nitrogen, amines, and hydrogen sulfide groups (Hanula et al. 2022a). Since the control sample did not contain any preservative additives and had the highest microbial load during the storage period, the fastest increase in pH over time was observed in this sample. At the end of the storage period, the highest pH was for the control sample, and burgers containing 2.7% and 1.8% PO‐TE nanocapsules had the lowest pH, and no statistically significant difference was observed between the two treatments on this day. Badar et al. (2023) also observed an increase in the pH of buffalo meat burgers during a 12‐day storage period at refrigerated temperature. Barros et al. (2021) reported that the combination of wheat germ oil and algae nanoemulsions had no significant effect on the pH of beef burgers. The lack of a significant effect of the combination of different levels of flaxseed oil on the pH of chicken sausage was also reported by Reddy et al. (2022).

**TABLE 5:** Comparison of pH, total volatile basic nitrogen (TVB‐N), peroxide, thiobarbituric acid (TBA) indexes, carbonyl, and cooking loss of beef burgers during the storage period.

Samples	Storage time (day)	pH	TVB‐N (mg N/100 g)	Peroxide (meq/kg)	TBA (mg MDA/kg)
C	0	5.82 ± 0.03^E,a^	9.16 ± 0.18^E,a^	0.48 ± 0.02^E,a^	0.26 ± 0.02^E,a^
	3	5.89 ± 0.01^D,a^	12.58 ± 0.29^D,a^	1.02 ± 0.04^D,a^	0.41 ± 0.01^D,a^
	6	5.96 ± 0.01^C,a^	20.59 ± 0.14^C,a^	1.75 ± 0.02^C,a^	0.85 ± 0.02^C,a^
	9	6.10 ± 0.02^B,a^	27.46 ± 0.33^B,a^	2.89 ± 0.05^B,a^	1.30 ± 0.03^B,a^
	12	6.21 ± 0.01^A,a^	33.10 ± 0.27^A,a^	4.00 ± 0.04^A,a^	1.97 ± 0.01^A,a^
N0.9%	0	5.83 ± 0.02^D,a^	9.23 ± 0.24^E,a^	0.47 ± 0.03^E,a^	0.26 ± 0.01^E,b^
	3	5.85 ± 0.02^D,b^	10.37 ± 0.20^D,b^	0.76 ± 0.02^D,b^	0.33 ± 0.03^D,b^
	6	5.91 ± 0.00^C,b^	16.01 ± 0.37^C,b^	1.23 ± 0.02^C,b^	0.61 ± 0.01^C,b^
	9	5.98 ± 0.01^B,b^	22.54 ± 0.28^B,b^	1.94 ± 0.01^B,b^	0.86 ± 0.03^B,b^
	12	6.03 ± 0.02^A,b^	26.52 ± 0.19^A,b^	3.18 ± 0.06^A,b^	1.44 ± 0.02^A,b^
N1.8%	0	5.85 ± 0.01^D,a^	9.27 ± 0.15^E,a^	0.43 ± 0.04^E,a^	0.24 ± 0.02^E,a^
	3	5.85 ± 0.01^D,b^	9.93 ± 0.26^D,c^	0.59 ± 0.03^D,c^	0.29 ± 0.02^D,bc^
	6	5.89 ± 0.00^C,c^	13.24 ± 0.23^C,c^	0.99 ± 0.03^C,c^	0.35 ± 0.02^C,c^
	9	5.96 ± 0.00^B,c^	17.30 ± 0.30^B,c^	1.51 ± 0.04^B,c^	0.62 ± 0.01^B,c^
	12	5.99 ± 0.01^A,c^	23.62 ± 0.24^A,c^	2.25 ± 0.03^A,c^	1.13 ± 0.03^A,c^
N2.7%	0	5.85 ± 0.02^D,a^	9.21 ± 0.26^D,a^	0.46 ± 0.03^E,a^	0.25 ± 0.02^D,a^
	3	5.86 ± 0.00^D,b^	9.56 ± 0.41^D,c^	0.61 ± 0.02^D,c^	0.26 ± 0.01^D,c^
	6	5.89 ± 0.01^C,c^	11.69 ± 0.33^C,d^	0.89 ± 0.02^C,d^	0.31 ± 0.01^C,d^
	9	5.93 ± 0.02^B,d^	14.16 ± 0.45^B,d^	1.14 ± 0.02^B,c^	0.48 ± 0.02^B,d^
	12	5.97 ± 0.01^A,c^	18.91 ± 0.39^A,d^	1.92 ± 0.04^A,c^	0.87 ± 0.02^A,d^

Samples	Storage time (day)	Carbonyl (nmol/mg protein)	Cooking loss (%)
C	0	2.71 ± 0.03^E,a^	18.48 ± 0.56^A,a^
	3	3.10 ± 0.06^D,a^	18.02 ± 0.32^A,a^
	6	3.96 ± 0.06^C,a^	18.75 ± 0.26^A,a^
	9	4.51 ± 0.09^B,a^	19.89 ± 0.60^A,a^
	12	5.18 ± 0.03^A,a^	19.00 ± 0.49^A,a^
N0.9%	0	2.73 ± 0.04^D,a^	17.47 ± 0.42^A,b^
	3	2.87 ± 0.01^D,b^	17.76 ± 0.37^A,b^
	6	3.24 ± 0.07^C,b^	17.23 ± 0.31^A,b^
	9	3.49 ± 0.03^B,b^	17.94 ± 0.48^A,b^
	12	3.90 ± 0.04^A,b^	17.18 ± 0.35^A,b^
N1.8%	0	2.70 ± 0.05^D,a^	17.43 ± 0.51^A,bc^
	3	2.84 ± 0.03^D,b^	17.59 ± 0.44^A,bc^
	6	3.11 ± 0.08^C,bc^	17.99 ± 0.37^A,bc^
	9	3.40 ± 0.02^B,c^	17.51 ± 0.30^A,b^
	12	3.78 ± 0.03^A,c^	17.25 ± 0.52^A,bc^
N2.7%	0	2.76 ± 0.03^D,a^	16.46 ± 0.35^A,c^
	3	2.81 ± 0.05^D,b^	16.61 ± 0.59^A,c^
	6	3.03 ± 0.04^C,c^	16.89 ± 0.41^A,c^
	9	3.28 ± 0.07^B,d^	16.14 ± 0.33^A,c^
	12	3.50 ± 0.03^A,d^	16.92 ± 0.38^A,c^

*Note:* Values represent mean (*n* = 3) ± SD Small and big different letters indicate significant difference among samples and storage period at 5% level of probability, respectively. C: control (without additive), N: Nanocapsules added to the burger formulation at levels of 0%, 0.9%, 1.8% and 2.7% (N0.9, N1.8 and N2.7) PO‐TE nanocapsules.

#### 3.4.2 TVB‐N

The TVB‐N test is used to measure volatile nitrogenous compounds produced by the activity of microorganisms and enzymes during storage. Trimethylamines are produced by the activity of spoilage bacteria, dimethylamines are produced by the activity of autolytic enzymes naturally present in meat, and ammonia is produced by the catabolism of nucleotides and deamination of amino acids. The production of volatile nitrogenous compounds is generally associated with the development of spoilage of protein‐rich food products (Li et al. 2012). The TVB‐N values of different burger treatments during storage are compared in Table 5. The incorporation of PO‐TE nanocapsules at the beginning of the storage period did not significantly affect the TVB‐N content of the burgers. The TVB‐N content of burgers increased significantly over time (*p* < 0.05), ranging from 9.16 to 9.27 mg N/100 g at the beginning of the storage period to 18.91–33.10 mg N/100 g on the 12th day. The increase in TVB‐N content of different burger treatments during storage is related to some reactions including nucleotide degradation, microbial activity, amine oxidation, and deamination of free amino acids (Tometri et al. 2020). Since the amount of TVB‐N is related to spoilage and growth of microorganisms and the antimicrobial activity of TE has been confirmed by previous researchers (Azizkhani et al. 2021; Socaciu et al. 2021), and in the present study, the incorporation of nanocapsules containing this essential oil into the burger formulation reduced the growth and activity of spoilage microorganisms and produced lower amounts of volatile nitrogen compounds in the enriched burgers compared to the control sample. At the end of the storage period, the highest amount of TVB‐N was obtained in the control sample, and with increasing the level of PO‐TE nanocapsules, the TVB‐N level of the burgers decreased significantly (*p* < 0.05), and the lowest amount of TVB‐N was observed on the last day in the burger enriched with 2.7% nanocapsule level. The maximum allowable level for TVB‐N in meat and meat products has been set at 25 mg N/100 g (Majdinasab et al. 2020), and the results obtained in the present study showed that the control sample until the sixth day, the burger containing 0.8% nanocapsule level until the ninth day, and the other treatments until the last day of storage had acceptable TVB‐N levels. In line with these results, Raeisi et al. (2021) also reported an increase in TVB‐N levels in chicken nuggets during the storage period and found that the incorporation of fish oil‐microencapsulated garlic essential oil into the nugget formulation reduced the intensity of the increase in TVB‐N levels in enriched nuggets compared to the control, and increasing the level of capsules also reduced TVB‐N levels. Hasani et al. (2020) also showed that during 18 days of refrigerated storage, the amount of TVB‐N in burgers increased, but the incorporation of microencapsulated lemon essential oil was able to significantly reduce the rate of production of volatile nitrogenous bases in enriched samples compared to the control.

#### 3.4.3 Oxidative Stability

The oxidative stability of burgers was investigated by measuring peroxide and TBA indices and the results are presented in Table 5. At the beginning of the storage period, the combination of different levels of PO–TE nanocapsules did not show a significant effect on the oxidative indices of burgers. Over time, the peroxide and TBA indices in burgers increased significantly (*p* < 0.05) due to the development of lipid oxidation and the production of higher amounts of primary and secondary products resulting from oxidation, so that the peroxide and TBA indices at the beginning of the storage period were in the range of 0.43–0.48 meq/kg and 0.24–0.26 mg MDA/kg, respectively, and on the twelfth day they reached 1.92–4.00 meq/kg and 0.87–1.97 mg MDA/kg, respectively. The highest rate of increase in these oxidative indices over time was observed in the control sample, so that at the end of the storage period, the highest level of these oxidative indices was obtained in the control sample and with increasing the level of PO–TE nanocapsules in the burger formulation, the levels of peroxide and TBA indices decreased significantly (*p* < 0.05).

Lipid oxidation is one of the important chemical processes responsible for the degradation and loss of quality of meat and meat products and reduces the shelf life of these products. In general, vegetable oils are rich in polyunsaturated fatty acids and are sensitive to lipid oxidation. The improvement of oxidative stability of burgers due to the incorporation of nanoemulsions can be related to several factors. Some vegetable oils contain high amounts of natural antioxidants such as alpha‐tocopherol, which can increase the ratio of antioxidants to peroxides in the product. The combination of purslane oil, rich in ω‐3 polyunsaturated fatty acids, with tarragon essential oil, a source of phenolic and aromatic antioxidants, provides a complementary antioxidant strategy in which lipid‐derived susceptibility to oxidation is counterbalanced by plant‐derived radical‐scavenging activity. Researchers also stated that PO contains phenolic compounds that exhibit antioxidant effects (Hamed et al. 2024). TE also has significant antioxidant activity (Azizkhani et al. 2021; Khajeh et al. 2024), and therefore can improve the oxidative stability of PO as well as the oxidative stability of the product. On the other hand, the microencapsulation process is an important and effective factor in improving the stability of oils (Alasalvar et al. 2022). The reasons stated can generally be a justification for improving the oxidative stability of beef burgers due to enrichment with PO–TE nanocapsules. As a result of increasing the nanocapsule level, due to the increase in the content of active and antioxidant compounds (especially phenolic compounds), the oxidation indices showed a significant decrease. The antioxidant activity of essential oils and plant extracts is often related to their phenolic compounds. These compounds have one or more aromatic rings and hydroxyl groups in their structure, which act as electron donors, reducing agents, metal ion chelators, and singlet oxygen neutralizers, and thus show significant antioxidant activity (Mumivand et al. 2017). Xiao and Ahn (2023) also stated that the use of combining fish oil with garlic essential oil improved the oxidative stability of this oil. Barros et al. (2021) also showed that the incorporation of wheat germ oil and algae nanoemulsions into beef burger formulations caused a significant reduction in the TBA index of the enriched burgers produced compared to the control, and the highest effect was related to the combined use of these oils. These researchers attributed their findings to the presence of high levels of α‐tocopherols in these oils. In another study by Barros et al. (2020), replacing part of the animal fat with tiger seed oil emulsion improved the oxidative stability and reduced the TBA index of enriched burgers compared to the control. An increase in the oxidative index of burgers during refrigerated storage was also reported in the study by Badar et al. (2023).

#### 3.4.4 Protein Oxidation

Since meat and meat products are rich sources of proteins, protein oxidation is effective in investigating their spoilage during storage. To investigate the severity of protein oxidation in protein‐rich products, the carbonyl number was calculated and the changes in its values in different burger treatments enriched with different levels of PO‐TE nanocapsules during a 12‐day storage period at refrigerated temperature are shown in Table 5. At the beginning of the storage period, the carbonyl values of different burger treatments were in the range of 2.70–2.76 nmol/mg protein, and the combination of different levels of nanocapsules did not show a significant effect on the carbonyl value of burgers. The carbonyl value of burgers increased significantly (*p* < 0.05) during the storage period due to the development of protein oxidation and reached 3.50–5.18 nmol/mg protein on the 12th day. The highest rate of increase in carbonyl content over time was observed in the control sample, so that at the end of the storage period, the highest carbonyl content was in the control sample and with increasing the level of PO‐TE nanocapsules in the burger formulation, the carbonyl content of the burgers decreased significantly (*p* < 0.05). Protein oxidation is directly related to lipid oxidation and lipid oxidation products lead to stimulation of protein oxidation; therefore, antioxidants are able to reduce the severity of protein oxidation (Ashrafi et al. 2024). In line with these results, dos Santos et al. (2024) also found that enrichment of fresh sausage using microencapsulated acai oil reduced protein oxidation in the produced samples. de Carvalho et al. (2019) also showed in their study that during the storage period, the carbonyl number of different treatments of lamb burgers containing chia oil emulsion increased, but the incorporation of guarana seed and pitanga leaf extracts into the formulation of this burger reduced the severity of protein oxidation over time compared to the control.

#### 3.4.5 Cooking Loss

The cooking loss values of different burger treatments enriched with different levels of PO‐TE nanocapsules are given in Table 5. At the beginning of the storage period, the control sample had the highest cooking loss (18.77%), and with the incorporation of PO‐TE nanocapsules and increasing its level in the burger formulation, the percentage of cooking loss decreased (*p* < 0.05), so that the lowest cooking loss on this day was related to the burger containing 2.7% nanocapsules (16.41%). Over time, a slight increase in the cooking loss of the burgers was observed, but this increase was not statistically significant. At the end of the storage period, the control sample had the highest cooking loss (19.32%) and the lowest was obtained in the burger containing 2.7% nanocapsules (16.61%). In general, the decrease in moisture content and fat percentage during the thermal process is the reason for the cooking loss of the product. The reduction in the percentage of cooking loss of burgers enriched with PO‐TE nanocapsules can be attributed to the use of GA and WPI to produce these nanocapsules. Because these biopolymers have a favorable water retention capacity (Zhang et al. 2022), and can reduce the amount of moisture loss of the product during the cooking process, thereby reducing the percentage of cooking loss. Also, due to the use of the encapsulation process, the release of oil from the capsules is slower and its loss during the cooking process is reduced (Heck et al. 2017). In agreement, Alasalvar et al. (2022) reported a significant reduction in the percentage of cooking loss of beef burgers due to the incorporation of hazelnut oil microcapsules. In line with these results, Badar et al. (2023) also achieved similar results and observed an improvement in the cooking efficiency of buffalo meat burgers due to the replacement of part of the fat in the formulation with hydrogel emulsions of peanut and walnut oils. The improvement in the cooking efficiency of sausages due to the increase in the level of flaxseed oil was also observed by Reddy et al. (2022). In the study of Hanula et al. (2022b), in agreement with the results of the present study, no significant change was observed in the cooking loss of burgers during storage.

#### 3.4.6 Fatty Acid Profile

The fatty acid profile of burger samples was determined by GC system and the fatty acids present in different burger treatments are presented in Table 6. In the control burger, oleic (43.55%), palmitic (22.34%), stearic (10.37%), and linoleic (7.17%) fatty acids were the dominant fatty acids, respectively, and in general, the highest contribution of fatty acids was related to MUFA (48.98%), SFA (35.02%), and PUFA (7.83%), respectively. By incorporating PO–TE nanocapsules into the beef burger formulation, no significant change was observed in the content of lauric acid, myristoleic acid, and behenic acid, but myristic, palmitic, stearic, and oleic fatty acids decreased, and the content of linoleic and linolenic fatty acids increased significantly (*p* < 0.05). Increasing the level of nanocapsule substitution in the formulation generally resulted in a decrease in SFA and MUFA content and a significant increase in PUFA content in the enriched burgers (*p* < 0.05). The PUFA/SFA ratio and n6/n3 ratio in the control burger were 0.22 and 10.86, respectively, and with increasing the level of substitution, the PUFA/SFA ratio increased significantly and the n6/n3 ratio decreased significantly (*p* < 0.05). Thus, the nutritional value of the burgers improved due to enrichment because higher SFA content and also the n6/n3 ratio lead to obesity in the consumer (Alasalvar et al. 2022). In line with these results, Badar et al. (2023) also showed that replacing part of the fat in the formulation with hydrogel emulsions of peanut and walnut oils significantly reduced the SFA content and significantly increased the PUFA content, but the trend in the change in MUFA content depended on the type of oil used, so that the amount of these fatty acids decreased with the incorporation of walnut oil emulsion and significantly increased with the incorporation of peanut oil emulsion. Rengifo et al. (2024) studied the enrichment of fish burgers using sacha inchi oil microcapsules and showed that burgers containing 3% of oil microcapsules had a higher PUFA content (α‐linolenic acid, eicosapentaenoic acid, and docosahexaenoic acid) than the control burger. dos Santos et al. (2024) showed that enrichment of sausage formulations with microencapsulated acai oil improved the fatty acid profile of the produced sausages. Barros et al. (2021) observed that by adding wheat germ oil nanoemulsion to beef burger formulations, SFA and MUFA content significantly decreased, but PUFA content significantly increased. These researchers also reported linoleic acid as the dominant PUFA fatty acid in control burgers and burgers enriched with oil nanoemulsions.

**TABLE 6:** Fatty acid profile (%) of beef burger treatments.

Fatty acids	C	N0.9%	N1.8%	N2.7%
Lauric acid (C12:0)	0.05 ± 0.02^a^	0.07 ± 0.01^a^	0.06 ± 0.03^a^	0.06 ± 0.02^a^
Myristic acid (C14:0)	2.24 ± 0.07^a^	1.97 ± 0.04^b^	1.73 ± 0.10^c^	1.48 ± 0.05^d^
Myristoleic acid (C14:1)	1.51 ± 0.07^a^	1.48 ± 0.10^a^	1.45 ± 0.06^a^	1.47 ± 0.04^a^
Palmitic acid (C16:0)	22.34 ± 0.14^a^	20.69 ± 0.21^b^	19.78 ± 0.17^c^	17.91 ± 0.26^d^
Palmitoleic acid (C16:1)	3.92 ± 0.09^a^	3.60 ± 0.05^b^	3.39 ± 0.08^c^	3.11 ± 0.12^d^
Stearic acid (C18:0)	10.37 ± 0.29^a^	8.42 ± 0.34^b^	7.27 ± 0.25^c^	5.97 ± 0.41^d^
Oleic acid (C18:1)	43.55 ± 0.48^a^	42.89 ± 0.37^b^	41.91 ± 0.52^c^	39.85 ± 0.33^d^
Linoleic acid (C18:2)	7.17 ± 0.06^d^	10.05 ± 0.09^c^	12.23 ± 0.05^b^	14.19 ± 0.14^a^
Linolenic acid (C18:3)	0.66 ± 0.03^d^	2.10 ± 0.11^c^	3.66 ± 0.08^b^	5.02 ± 0.04^a^
Behenic acid (C22:0)	0.02 ± 0.01^a^	0.03 ± 0.01^a^	0.03 ± 0.02^a^	0.02 ± 0.02^a^
SFA	35.02 ± 0.42^a^	31.18 ± 0.46^b^	28.87 ± 0.53^c^	25.44 ± 0.39^d^
MUFA	48.98 ± 0.44^a^	47.97 ± 0.29^b^	46.75 ± 0.36^c^	44.43 ± 0.28^d^
PUFA	7.83 ± 0.38^d^	12.15 ± 0.42^c^	15.89 ± 0.39^b^	19.21 ± 0.33^a^
PUFA/SFA	0.22 ± 0.03^d^	0.39 ± 0.03^c^	0.55 ± 0.05^b^	0.75 ± 0.02^a^
n6/n3	10.86 ± 0.08^a^	4.79 ± 0.13^b^	3.34 ± 0.06^c^	2.83 ± 0.10^d^

*Note:* Values represent mean (*n* = 3) ± SD. Different letters in each column represent significant difference at 5% level of probability among samples.

Abbreviations: C, control (without additive); MUFA, monounsaturated fatty acids; N, nanocapsules added to the burger formulation at levels of 0%, 0.9%, 1.8% and 2.7% (N0.9, N1.8 and N2.7) PO‐TE nanocapsules; PUFA, poly unsaturated fatty acids; SFA, saturated fatty acids.

#### 3.4.7 Instrumental Color

The color of the burgers was examined by a calorimeter and the color indices obtained are presented in Table 7. At the beginning of the storage period, the incorporation of different levels of nanocapsules had no significant effect on the color indices L*, a* and b* of the burgers, and the values of these color indices of the burgers on this day were in the range of 49.81–51.04, 14.13–14.24 and 15.45–15.67, respectively. Over time, the levels of L*, a* and b* indices of the burgers gradually decreased and the intensity of this decrease in the control sample was significantly higher than in the burgers enriched with PO–TE nanocapsules (*p* < 0.05) and the incorporation of these nanocapsules resulted in better preservation of the color of the burgers during the storage period at refrigerated temperature. Color is generally considered one of the important parameters of meat products and plays a significant role in the acceptance of the product by consumers. The decrease in the intensity of the red color of burgers during storage is due to the oxidation of iron and the pigment oxymyoglobin and its conversion to the brown pigment metmyoglobin (Zamuz et al. 2018). The darkening of the color of burgers during storage can probably be attributed to the decrease in surface moisture, the formation of metmyoglobin, and the decrease in light refraction (Amiri et al. 2019). In agreement, Mokhtar and Eldeep (2020) also reported a decrease in the color indices L*, a*, and b* of beef burgers during storage at refrigerated temperature. The decrease in the intensity of the red color of burgers during storage due to the production of metmyoglobin was also observed by de Carvalho et al. (2019). In a study conducted by Barros et al. (2021), the combination of wheat germ oil and algae nanoemulsions did not show a significant effect on the color indices of beef burgers. In another study, the addition of Echium oil did not affect the color of sausages (Pires et al. 2019).

**TABLE 7:** Comparison of color indices of beef burgers during the storage period.

Samples	Storage time (day)	L*	a*	b*
C	0	49.81 ± 0.92^A,a^	14.24 ± 0.27^A,a^	15.45 ± 0.31^A,a^
	3	49.27 ± 1.05^A,a^	13.60 ± 0.15^B,b^	15.36 ± 0.23^A,a^
	6	48.53 ± 1.11^AB,b^	12.85 ± 0.22^C,b^	14.92 ± 0.18^B,b^
	9	47.41 ± 0.80^bc,a^	11.94 ± 0.29^D,c^	14.34 ± 0.35^C,b^
	12	46.16 ± 0.83^C,b^	11.33 ± 0.19^E,c^	13.85 ± 0.26^C,b^
N0.9%	0	50.27 ± 0.86^A,a^	14.19 ± 0.26^A,a^	15.51 ± 0.21^A,a^
	3	50.10 ± 0.90^AB,a^	14.00 ± 0.21^AB,a^	15.43 ± 0.16^AB,a^
	6	49.69 ± 0.88^AB,ab^	13.64 ± 0.16^B,a^	15.30 ± 0.23^AB,ab^
	9	48.96 ± 0.93^AB,ab^	13.18 ± 0.10^C,b^	15.10 ± 0.29^AB,a^
	12	48.27 ± 0.96^B,a^	12.69 ± 0.24^D,b^	14.89 ± 0.18^B,a^
N1.8%	0	50.81 ± 1.04^A,a^	14.20 ± 0.21^A,a^	15.54 ± 0.17^A,a^
	3	50.77 ± 0.94^A,a^	14.06 ± 0.16^A,a^	15.52 ± 0.24^A,a^
	6	50.53 ± 1.14^A,ab^	13.83 ± 0.29^AB,a^	15.39 ± 0.31^AB,ab^
	9	49.91 ± 1.05^A,a^	13.52 ± 0.24^bc,ab^	15.24 ± 0.19^AB,a^
	12	49.38 ± 0.90^A,a^	13.18 ± 0.17^C,a^	15.01 ± 0.32^B,a^
N2.7%	0	51.04 ± 1.13^A,a^	14.13 ± 0.18^A,a^	15.67 ± 0.30^A,a^
	3	51.10 ± 0.79^A,a^	14.05 ± 0.19^AB,a^	15.63 ± 0.26^A,a^
	6	50.96 ± 0.95^A,a^	13.91 ± 0.14^AB,a^	15.55 ± 0.29^A,a^
	9	50.27 ± 1.10^A,a^	13.69 ± 0.21^B,a^	15.31 ± 0.24^A,a^
	12	49.82 ± 1.02^A,a^	13.21 ± 0.15^C,a^	15.17 ± 0.30^A,a^

*Note:* Values are expressed as mean (*n* = 3) ± standard deviation. Different lowercase letters within a row indicate significant differences among treatments (*p* < 0.05).

Abbreviations: *a*, red–green; *b*, yellow–blue; C, control (without additive); *L, lightness*; N, nanocapsules added to the burger formulation at levels of 0%, 0.9%, 1.8% and 2.7% (N0.9, N1.8 and N2.7) PO‐TE nanocapsules; PO–TE, purslane seed oil–tarragon essential oil.

#### 3.4.8 Textural Parameters

Textural parameters of different burger treatments including hardness, cohesiveness, chewiness and springiness are given in Table 8. At the beginning of the storage period, the lowest hardness and chewiness were related to the control sample (11.03 N and 2.94 N mm, respectively), and the combination of different levels of PO–TE nanocapsules increased the hardness and chewiness of the burger texture (11.41–12.32 N and 3.07–3.51 N mm, respectively), but these changes were not significant at the 0.8% nanocapsule level compared to the control sample. Over time, the hardness and chewiness of the burgers gradually increased (*p* < 0.05), and the highest rate of increase was related to the control sample. At the end of the storage period, the control sample had the highest firmness and chewiness (22.65 N and 6.86 N mm, respectively), and there was no statistically significant difference between the hardness values of burgers containing 1.8% and 2.7% nanocapsule levels and the chewiness of all burgers containing different levels of nanocapsules on this day. At the beginning of the storage period, the values of the burgers' texture cohesiveness and springiness were in the range of 0.31–0.36 and 0.61–0.65 mm, respectively, and there was no statistically significant difference between the different treatments. No significant change was observed in the degree of cohesiveness and springiness of burgers' texture over time. Research has shown that as the fat percentage of the product decreases, a stronger protein network is formed, which can lead to hardness and chewiness of the meat product. The reduction of fat content in burgers due to substitution with vegetable oils has been observed by previous researchers (Alasalvar et al. 2022; Heck et al. 2019). On the other hand, the texture of PO–TE nanocapsule powder is completely different from animal fat, which could be the main reason for the changes in burger texture due to the replacement of part of the fat in the formulation with oil nanocapsules. In line with these results, Rengifo et al. (2024) also showed, similarly to the results of the present study, that enrichment of fish burgers with sacha inchi oil microcapsules increased the texture hardness and chewiness of the enriched burgers, but had no significant effect on the amount of cohesiveness and springiness. An increase in hardness and chewiness of burgers during storage was also observed in the study conducted by Hanula et al. (2022a, 2022b). Barros et al. (2021) enriched a beef burger formulation using wheat germ oil and algae nanoemulsions and observed that the use of these oil nanoemulsions increased the textural parameters of the produced burgers compared to the control.

**TABLE 8:** Comparison of textural parameters of beef burgers during the storage period.

Samples	Storage time (day)	Hardness (N)	Cohesiveness	Chewiness (N mm)	Springiness (mm)
C	0	11.03 ± 0.29^E,c^	0.35 ± 0.02^A,a^	2.94 ± 0.16^E,b^	0.61 ± 0.03^A,a^
	3	13.46 ± 0.42^D,a^	0.34 ± 0.03^A,a^	3.41 ± 0.23^D,a^	0.60 ± 0.02^A,a^
	6	16.10 ± 0.31^C,a^	0.34 ± 0.02^A,a^	3.96 ± 0.11^C,a^	0.57 ± 0.02^A,a^
	9	19.73 ± 0.49^B,a^	0.32 ± 0.02^A,a^	4.92 ± 0.19^B,a^	0.58 ± 0.02^A,a^
	12	22.65 ± 0.37^A,a^	0.33 ± 0.04^A,a^	6.86 ± 0.24^A,a^	0.55 ± 0.04^A,a^
N0.9%	0	11.41 ± 0.35^D,bc^	0.36 ± 0.03^A,a^	3.07 ± 0.25^D,ab^	0.61 ± 0.04^A,a^
	3	12.00 ± 0.32^D,c^	0.36 ± 0.02^A,a^	3.26 ± 0.22^CD,b^	0.60 ± 0.03^A,a^
	6	13.95 ± 0.28^C,b^	0.35 ± 0.02^A,a^	3.54 ± 0.18^bc,b^	0.58 ± 0.03^A,a^
	9	15.05 ± 0.44^B,b^	0.36 ± 0.03^A,a^	3.83 ± 0.12^B,b^	0.56 ± 0.03^A,a^
	12	17.01 ± 0.55^A,b^	0.34 ± 0.01^A,a^	4.51 ± 0.17^A,b^	0.55 ± 0.02^A,a^
N1.8%	0	11.87 ± 0.21^D,ab^	0.33 ± 0.03^A,a^	3.34 ± 0.14^D,a^	0.62 ± 0.02^A,a^
	3	12.24 ± 0.39^CD,bc^	0.33 ± 0.03^A,a^	3.50 ± 0.18^CD,ab^	0.60 ± 0.03^A,a^
	6	12.93 ± 0.42^bc,c^	0.33 ± 0.04^A,a^	3.69 ± 0.20^bc,ab^	0.61 ± 0.02^A,a^
	9	13.53 ± 0.36^B,c^	0.35 ± 0.02^A,a^	3.90 ± 0.16^B,b^	0.60 ± 0.02^A,a^
	12	14.93 ± 0.40^A,c^	0.33 ± 0.01^A,a^	4.40 ± 0.13^A,b^	0.58 ± 0.04^A,a^
N2.7%	0	12.32 ± 0.44^D,a^	0.31 ± 0.02^A,a^	3.51 ± 0.19^C,a^	0.65 ± 0.04^A,a^
	3	12.71 ± 0.25^D,b^	0.33 ± 0.02^A,a^	3.66 ± 0.11^C,ab^	0.65 ± 0.05^A,a^
	6	13.45 ± 0.27^C,c^	0.32 ± 0.01^A,a^	3.82 ± 0.13^bc,ab^	0.63 ± 0.04^A,a^
	9	14.29 ± 0.41^B,c^	0.33 ± 0.02^A,a^	4.11 ± 0.27^AB,b^	0.60 ± 0.03^A,a^
	12	15.41 ± 0.33^A,c^	0.34 ± 0.03^A,a^	4.52 ± 0.21^A,b^	0.60 ± 0.03^A,a^

*Note:* Values represent mean (*n* = 3) ± SD Small and big different letters indicate significant difference among samples and storage period at 5% level of probability, respectively.

Abbreviations: C, control (without additive); N, nanocapsules added to the burger formulation at levels of 0%, 0.9%, 1.8% and 2.7% (N0.9, N1.8 and N2.7) PO‐TE nanocapsules.

#### 3.4.9 Microbial Load

**FIGURE 4:**
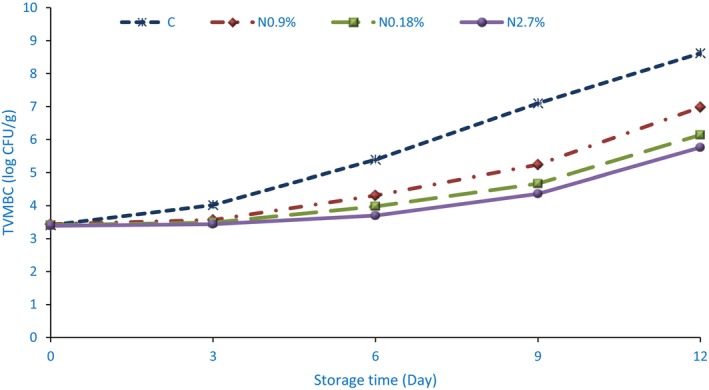
Changes in TVMBC (log CFU/g) of beef burgers during the storage period. C, control (without additive); N, nanocapsules added to the burger formulation at levels of 0%, 0.9%, 1.8% and 2.7% (N0.9, N1.8 and N2.7) PO‐TE nanocapsules; TVMBC, total viable mesophilic bacteria count.

The changes in TVMBC of different burger treatments enriched with different levels of PO–TE nanocapsules during a 12‐day storage period at refrigerated temperature are shown in Figure 4. At the beginning of the storage period, the incorporation of different levels of nanocapsules did not show a significant effect on the microbial load of the burgers. Over time, the TVMBC of the burgers increased significantly (*p* < 0.05) due to the growth and proliferation of bacteria, so that their number was in the range of 3.39–3.44 log CFU/g at the beginning of the storage period and reached 5.76–8.62 log CFU/g on the 12th day. The highest growth and proliferation rate of bacteria over time was related to the control sample, so that at the end of the storage period, the highest TVMBC was observed in this sample and, as expected, with increasing the level of PO–TE nanocapsules in the burger formulation, due to the increased content of bioactive compounds and increased antimicrobial effect, the number of these microorganisms in the burgers decreased significantly (*p* < 0.05). Plant essential oils are active agents with antimicrobial effect. Essential oils have a hydrophobic nature and this causes lipid separation in the cell membrane and mitochondria of bacteria and increases the permeability of the cell membrane. On the other hand, it has been stated that the chemical components of essential oils are able to adhere to the cell surface and destroy the bacterial cell wall (Munda et al. 2019). In general, various mechanisms have been stated for the antimicrobial function of essential oils. Essential oils initially destroy the stability of the overall structure of the bacterial cell, leading to the breakdown of the cell membrane surface and increased membrane permeability, leading to the leakage of intracellular contents to the outside, the destruction of ATP and the proton pump, and disrupting the metabolism of the bacterial cell (Hou et al. 2022). PO also has antimicrobial activity, and its activity has been confirmed in the research of Hamed et al. (2024) and Petropoulos et al. (2021). In general, it has been stated that omega‐3 fatty acids have antibacterial activity and exert this activity by disrupting the electron transport chain, inhibiting enzyme activity, uncoupling oxidative phosphorylation, stimulating autoxidation, and reducing nutrient absorption (Kondakova et al. 2019). On the other hand, unsaturated fatty acids have an amphipathic structure and interact with the bacterial cell membrane and inhibit bacterial growth (Hamed et al. 2024). Ebrahimi et al. (2022) also observed the effect of olive oil on reducing TVMBC of beef burgers in their study. The significant effect of TE in reducing the microbial load of chicken fillets during refrigerated storage was also reported in the study of Rahmani et al. (2020).

#### 3.4.10 Sensory Evaluation

**FIGURE 5:**
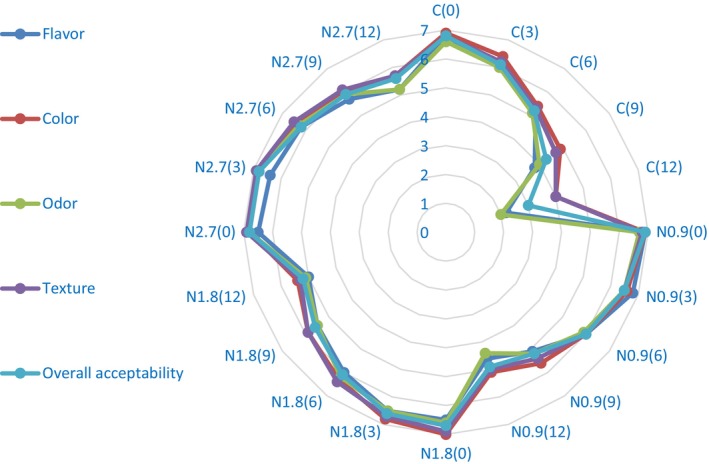
Comparison of sensory characteristics scores of beef burgers during the storage period. C, control (without additive); N, nanocapsules added to the burger formulation at levels of 0%, 0.9%, 1.8% and 2.7% (N0.9, N1.8 and N2.7) PO–TE nanocapsules. Numbers in parentheses represent storage days.

The results of statistical analysis of the data showed that various sensory properties of beef burgers including flavor, color, odor, texture and overall acceptance were significantly affected by the addition of nanocapsules and storage time (*p* < 0.05). The changes in the mean scores of flavor, color, odor, texture and overall acceptability of different burger treatments enriched with different levels of PO‐TE nanocapsules during the refrigerated storage period are shown in Figure 5. At the beginning of the storage period, the incorporation of different levels of PO‐TE nanocapsules did not show a significant effect on the sensory properties of burgers and the most important reason for this could be the use of the encapsulation process, one of the main advantages of which is to mask the strong organoleptic properties of the active ingredients. Over time, from the beginning to the end of the storage period, a gradual decrease in the sensory scores of the burgers was observed, which, as expected, occurred significantly faster in the control sample than in the enriched burgers due to the lack of natural preservatives (*p* < 0.05). In general, the decrease in the sensory scores of the burgers over time is related to spoilage‐related reactions. Due to microbial growth and production of secondary metabolites and secretion of enzymes and breakdown of proteins, off‐flavors and odorous compounds are created and also affect the texture of the product. Another destructive reaction over time is the oxidation of lipids and proteins, which has a negative and undesirable effect on the sensory properties of the product. The decrease in moisture content and texture hardness over time can also be another reason for the decrease in product acceptance by consumers. As expected, due to the significant antioxidant and antimicrobial activity of PO‐TE nanocapsules, the incorporation of this nanocapsule into the formulation resulted in better preservation of the sensory acceptability of the enriched burgers during the refrigerated storage period, with the greatest effect being associated with the higher levels (1.8% and 2.7%), such that these enriched burgers were acceptable to the evaluators by the end of the 12‐day refrigerated storage period. Rengifo et al. (2024) similarly showed that enrichment of fish burgers with sacha inchi oil microcapsules did not have an adverse effect on the sensory properties of the produced burgers. Rahmani et al. (2020) also stated that coating chicken fillets with active coatings containing 2% TE improved the sensory acceptability of chicken fillets. Alasalvar et al. (2022) also showed, in agreement with the results of the present study, that replacing part of the fat in the beef burger formulation with hazelnut oil microcapsules did not have a significant effect on the sensory properties of the enriched burgers produced compared to the control sample. Although every effort was made to standardize testing conditions, sensory evaluation inherently involves subjective perception, which may introduce inter‐individual variability. The relatively small panel size and short‐term testing period also represent limitations that could be addressed in future studies through larger, more diverse panels and repeated testing sessions.

## 4 Conclusion

The study found that PO‐TE capsules, with their nanometer size, high oil encapsulation efficiency, and desirable zeta potential, were stable. During refrigerated storage, the pH, TVB‐N, oxidation indices, carbonyl number, and microbial load of burgers increased significantly, with the highest rate being related to the control sample. Enrichment with PO‐TE nanocapsules improved antioxidant and antimicrobial activity, delayed spoilage, and maintained the color of the burgers. The incorporation of PO‐TE nanocapsules reduced SFA and MUFA content and increased PUFA content in enriched burgers. The PUFA/SFA ratio increased and the n6/n3 ratio decreased, improving the nutritional value of the burger. PO‐TE nanocapsules also maintained the sensory properties of the burger during refrigerated storage. The encapsulation of bioactive compounds using gum Arabic and whey protein isolate has shown effectiveness in enhancing stability and antimicrobial performance. However, it faces limitations such as partial loss of volatile components, particle size heterogeneity, and lower encapsulation efficiency of highly hydrophobic cores. Scaling up for industrial production may face challenges in equipment cost, process optimization, and product uniformity. PO‐TE nanocapsules enhance beef burger quality, but further research is needed for other burger types. On the other hand, the study found that PO‐TE nanocapsules have significant preservative potential, but it did not compare them with other conventional food preservatives like synthetic antioxidants or natural bio‐preservatives. Future research should include systematic comparisons with both synthetic and plant‐derived preservatives to assess their efficacy, cost‐effectiveness, and consumer acceptance.

## Author Contributions

**Mozhgan Mehrabi:** formal analysis, software application, preparation of the original draft. **Homa Baghaei:** data curation, supervision, investigation, manuscript revision. **Majid Mohammadhosseini:** data curation, investigation, validation. **Fariborz Nahidi:** investigation, data review, manuscript revision.

## Funding

The authors have nothing to report.

## Ethics Statement

The authors have nothing to report.

## Consent

The authors have nothing to report.

## Conflicts of Interest

The authors declare no conflicts of interest.

## Data Availability

The datasets generated during and/or analyzed during the current study are available from the corresponding author on reasonable request.
